# Safety and efficacy of surgical treatment for brainstem hemangioblastoma: a meta-analysis

**DOI:** 10.1007/s10143-020-01305-3

**Published:** 2020-04-30

**Authors:** Xiangdong Yin, Chunwei Li, Liang Li, Hongzhou Duan

**Affiliations:** Department of Neurosurgery, Peking University First Hospital, Peking University, No. 8 Xishiku Street, Xicheng District, Beijing, China

**Keywords:** Brainstem, Hemangioblastoma, Surgical treatment, Meta-analysis

## Abstract

Brainstem hemangioblastomas are benign, highly vascular tumors located in the mesencephalon, pons, and medulla oblongata. Although surgical resection is currently considered the main therapeutic option for symptomatic lesions, evidence supporting the application of microsurgery has not been systematically assessed. This meta-analysis aims to evaluate the safety and efficacy of surgical treatment for brainstem hemangioblastomas. A comprehensive search of the PubMed, Embase, and Web of Science databases was performed to identify all English language publications reporting the outcomes of surgical treatment for brainstem hemangioblastomas. Studies from January 1990 to July 2019 with ≥ 10 cases were included. We analyzed the surgical outcomes, including gross total resection, mortality, neurological morbidity, and functional outcome according to the McCormick Scale or Karnofsky Performance Scale. Thirteen studies with 473 cases were included. The pooled proportion of gross total resection was 98% (95% confidence interval (CI), 94–100%). Overall mortality and neurological morbidity were 4 (95% CI, 2–6%) and 13% (95% CI, 7–20%), respectively. Favorable functional outcomes at the last follow-up were achieved in 85% (95% CI, 78–92%) of all patients. Improved or stable functional outcomes at long-term follow-up were achieved in 94% (95% CI, 89–97%) of patients. This meta-analysis revealed that surgical treatment for brainstem hemangioblastomas is technically feasible and effective with lasting patient benefits and cure.

**Electronic supplementary material:**

The online version of this article (10.1007/s10143-020-01305-3) contains supplementary material, which is available to authorized users.

## Introduction

Hemangioblastomas are relatively rare and histologically benign neoplasms, which may occur either sporadically or in association with von Hippel-Lindau disease (VHL) [16]; they usually grow slowly and account for 1.5–3.7% of intracranial tumors [3, 18, 23, 33] and 3–4% of spinal tumors [3, 18]. Hemangioblastomas of the central nervous system (CNS) are most commonly located in the cerebellum, followed by the spinal cord and brainstem [32].

To date, therapeutic options include radiosurgery and microsurgical resection of the tumor. Radiosurgery may cause severe radiation-induced adverse effects and is only appropriate for inoperable, residual, or recurrent hemangioblastomas [29]. Thus, microsurgical resection of tumors is considered the optimal option for patients with symptomatic or progressive CNS hemangioblastoma [7, 9, 24].

Brainstem hemangioblastomas are defined as tumors originating from the mesencephalon, pons, and medulla oblongata; they account for 5–15% of all intracranial hemangioblastomas [6, 12]. The complex anatomical structures and pivotal neural structures of the brainstem create a higher risk for morbidity and mortality. Consequently, surgical treatment for brainstem hemangioblastoma poses a significant challenge for neurosurgeons. To date, there have been many studies on the surgical outcomes of microsurgical resection of brainstem hemangioblastomas [4, 10, 13, 15, 17, 22, 33–38, 40]. We conducted a meta-analysis of surgical outcomes from the published literature to evaluate the safety and efficacy of surgical treatment for brainstem hemangioblastoma in this study.

## Materials and methods

We conducted a meta-analysis following the guidelines of the Preferred Reporting Items for Systematic Reviews and Meta-Analyses (PRISMA) [20].

### Study selection

We performed a comprehensive literature review using appropriate medical subject heading (MeSH) terms and the corresponding random words, including“brainstem”, “pons”, “mesencephalon”, “medulla oblongata”, “hemangioblastoma”, “von Hippel-Lindau disease”, “surgery”, “microsurgery”, “neurosurgery”, and “craniotomy”, in both “AND” and “OR” combinations to search the PubMed, Embase, and Web of Science databases. The search strategy can be found in Online Resource 1.

### Inclusion and exclusion criteria

Studies were included if they (1) were published from January 1990 to July 2019; (2) reported extent of tumor resection; (3) reported postoperative mortality; and (3) reported the preoperative and postoperative functional status. Exclusion criteria were as follows: non-English studies, case reports, review articles, conference abstracts, technical notes, operative video, articles that reported less than 10 cases, and studies lacking critical data.

### Data extraction

Extracted data included the first author’s name, publication year, country, patient demographics, tumor locations, and the total numbers of cases. We studied the surgical outcomes, including extent of tumor resection, postoperative mortality, and preoperative and postoperative functional status in the hospital and at long-term follow-up. Assessment of functional status was based on the McCormick Scale [19] or Karnofsky Performance Scale (KPS) [26]. Neurological morbidity was defined as the proportion of patients experiencing deterioration of short-term functional status, which was usually assessed immediately after surgery or at discharge.

### Quality assessment

The quality of all the included studies was graded using the methodological index for non-randomized studies (MINORS) [28]. The assessment of study quality was based on the checklist of 8 items for noncomparative studies, and the evaluation was scored 0 (not reported), 1 (reported but inadequate), or 2 (reported and adequate) for each item. Two authors (Yin and Li) graded the studies independently. Disagreement was solved by discussion.

### Statistical analysis

The meta-analyses were performed in the statistical software R version 3.6.1 (R Foundation for Statistical Computing, Vienna, Austria) [1] using the meta-packages [2]. The Freeman-Tukey double arcsine method was used for the transformation of surgical outcomes of each study prior to calculating the pooled results [8]. We used random-effects model in all the analyses. Between-study heterogeneity was assessed with Cochran’s Q test [5]. A *P* value for the test of heterogeneity < 0.10 was considered evidence of heterogeneity [27]. The *I*^2^ statistic, which describes the percentage of variance due to heterogeneity, was also considered. Heterogeneity across all the studies was classified into low, moderate, and high with *I*^2^ values of 25, 50, and 75%, respectively [11]. Egger’s linear regression test was applied to assess potential publication bias. A *P* value < 0.05 for the Egger’s test was considered statistically significant [30].

## Results

**Fig. 1:**
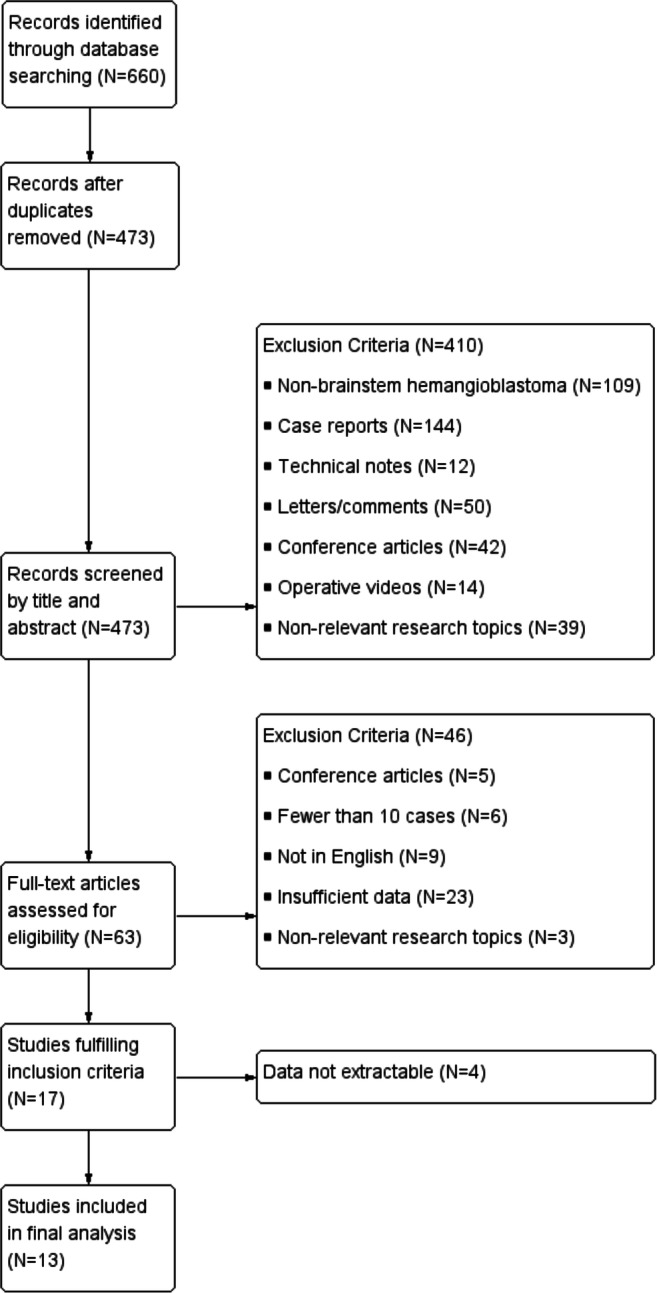
Flow chart for the study search and selection

The search initially yielded 694 studies. A total of 473 studies were included after the removal of duplicates. On initial abstract and title review, we removed 144 case reports, 50 letters or article comments, 42 conference articles, 39 articles reporting non-relevant research topics, 14 articles about operative videos, 12 technical notes, and 109 studies reporting hemangioblastomas in other locations. After carefully screening the remaining studies, we excluded 23 studies without sufficient data, 9 non-English studies, 6 studies with fewer than 10 cases, 5 conference articles, and 3 studies reporting non-relevant research topics. Four of the 17 remaining studies fulfilling inclusion criteria were removed for failing to provide extractable data. Thus, a total of 13 identified studies were included in this meta-analysis (Fig. 1) [4, 10, 13, 15, 17, 22, 33–38, 40]. Details of the included studies are shown in Table 1.

**Table 1:** Baseline clinical characteristics of studies included in the meta-analysis

Author, year	Country	Type of study	No. of patients	Mean age (year)	Females/males	Diameter of tumor (mm)	Cyst formation	VHL-related lesions/percentage
Chen, 2013	China	Retro	24	37.2	8/16	36	6	NR/NR
Giammattei, 2016	France	Retro	31	33	15/16	13	24	25/80.6%
Joseph, 2018	India	Retro	27	29	11/16	33.4	16	6/22.2%
Liu, 2015	China	Retro	62	35.6	26/36	29	19	17/27.4%
Ma, 2015	China	Retro	116	28	56/60	18	17	13/11.2%
Pavesi, 2010	Italy	Retro	14	34	9/4	NR	12	14/100%
Wang, 2001	China	Retro	47	35.2	18/29	NR	39	4/8.5%
Weil, 2003	USA	Retro	12	31.7	6/6	NR	9	12/100%
Wind, 2011	USA	Retro	44	35.7	27/17	NR	33	44/100%
Wu, 2013	China	Retro	11	42.27	4/7	40	0	NR/NR
Xu, 2010	China	Retro	18	33.4	5/13	26	5	2/11.1%
Yin, 2014	China	Retro	34	38	20/14	NR	12	NR/NR
Zhou, 2005	China	Retro	33	45	16/17	NR	4	0/0%

*NR* not reported

### Baseline characteristics

All 13 studies were retrospective and observational. A total of 473 patients with brainstem hemangioblastomas were presented, of whom 221 were females (46.7%) and 252 were males (53.3%). The mean age of the included patients at surgery was 34.0 years (range 6–71 years). Forty-one percent of the hemangioblastomas were associated with cyst formation. The size of the tumors or cysts was reported in 7 studies [4, 10, 13, 15, 17, 36, 37], and the mean diameter was 21.3 mm (range 2–56 mm). In total, 39.6% of the tumors were related to VHL disease [10, 13, 15, 17, 22, 33–35, 37, 40]. The most common region involved was the medulla oblongata (55.0%). According to the relationship between the tumor and surrounding parenchyma, 15.1% of brainstem hemangioblastomas were entirely intrabrainstem or intramedullary [4, 10, 13, 15, 17, 34]. Details of the study characteristics are shown in Table 2.

**Table 2:** Meta-analysis of the surgical outcomes of brainstem hemangioblastomas

	Events	Total	Proportion (95% CI)	Model^a^	Test of heterogeneity^b^		Egger’s test^c^ (*P* value)
					*I*^2^	*P* value	
Gross total resection	451	473	98% (94–100%)	R	71%	< 0.01	0.97
Postoperative mortality	23	473	4% (2–6%)	R	0%	0.47	0.87
Neurological morbidity	48	323	13% (7–20%)	R	57%	0.02	0.63
Favorable functional outcomes	200	237	85% (78–92%)	R	50%	0.05	0.68
Improved or stable functional outcomes	325	349	94% (89–97%)	R	51%	0.03	0.20

^a^R for random-effects model

^b^*P* < 0.10 is considered evidence of heterogeneity; *I*^2^ is interpreted as the percentage of variance due to heterogeneity

^c^Egger’s test to evaluate publication bias, *P* < 0.10 is considered statistically significant

### Extent of tumor resection

**Fig. 2:**
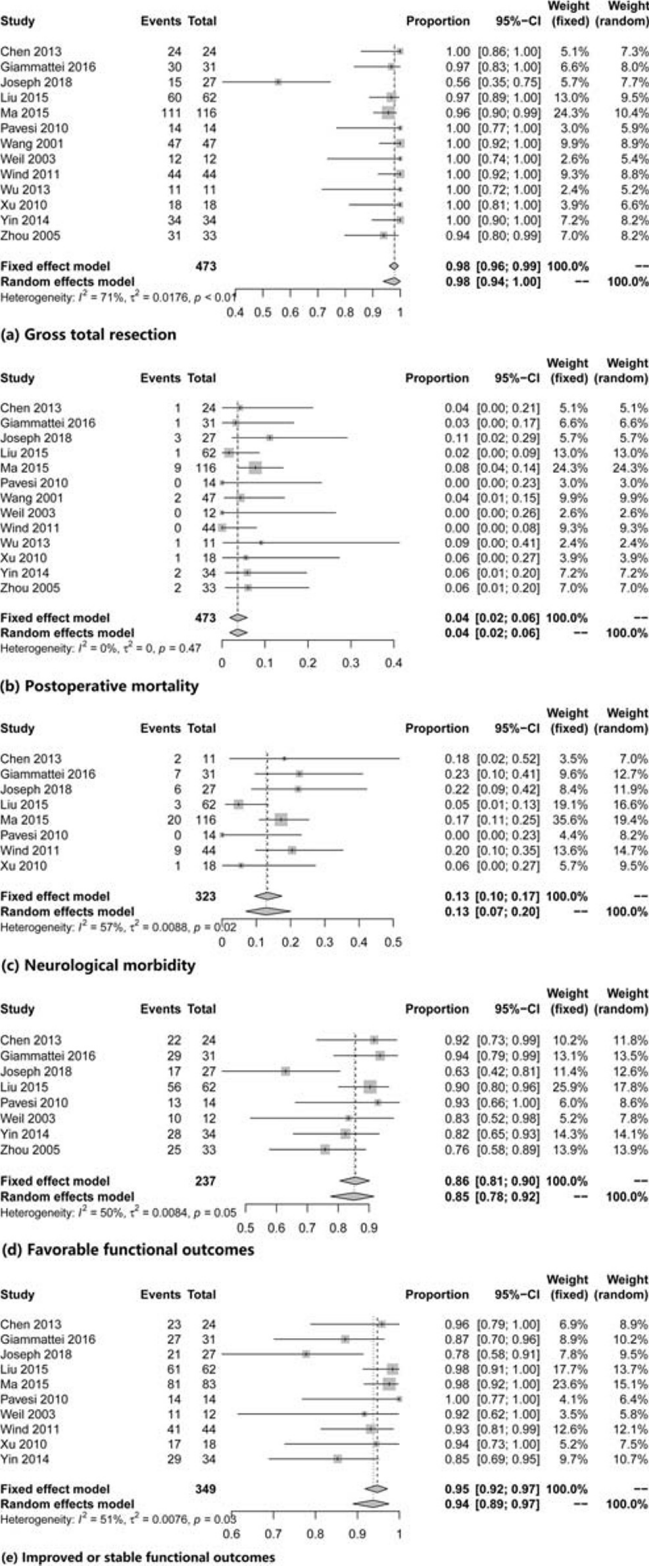
Forest plots showing the surgical outcomes including **a** gross total resection, **b** postoperative mortality, **c** neurological morbidity, **d** favorable outcomes at last follow-up, and **e** improved or stable functional outcomes compared with preoperative condition

All 13 included articles provided complete information on tumor resection that could be extracted and analyzed [4, 10, 13, 15, 17, 22, 33–38, 40]. The extent of tumor resection was confirmed by postoperative computed tomography (CT) or magnetic resonance imaging (MRI). Gross total resection was achieved in 98% (95% CI, 94–100%; *I*^2^ = 71%) of the cases. The heterogeneity between the groups was significant (*I*^2^ = 71%, *P* < 0.01) (Fig. 2 and Table 2).

### Postoperative mortality and morbidity

Surgical mortality was reported in all the included articles [4, 10, 13, 15, 17, 22, 33–38, 40]. The results showed that the pooled proportion of surgical mortality was 4% (95% CI, 2–6%; *I*^2^ = 0%). Heterogeneity testing revealed no evidence of heterogeneity between all the groups (*I*^2^ = 0%, *P* = 0.47) (Fig. 2 and Table 2).

Data on neurological morbidity were analyzed in 8 of 13 studies [4, 10, 13, 15, 17, 22, 35, 37]. The cumulative proportion of morbidity was 13% (95% CI, 7–20%; *I*^2^ = 57%). There was evident heterogeneity between the groups (*I*^2^ = 57%, *P* = 0.02) (Fig. 2 and Table 2).

### Functional outcomes at long-term follow-up

Functional outcomes at long-term follow-up were reported in 8 studies [4, 10, 13, 15, 22, 34, 38, 40]. A favorable functional status was determined if the patient was self-independent with grade I/II on the McCormick Scale or KPS ≥ 80. The pooled results showed that a favorable functional outcome was achieved in 85% (95% CI, 78–92%; *I*^2^ = 50%) of all patients at long-term follow-up. The heterogeneity between the groups was relatively moderate (*I*^2^ = 50%; *P* = 0.05) (Fig. 2 and Table 2).

We also compared postoperative functional status at the last follow-up compared with the preoperative functional condition. Data were obtained from 10 studies [4, 10, 13, 15, 17, 22, 34, 35, 37, 38]. The pooled analysis showed that improved or stable functional outcomes at the last follow-up compared with the preoperative function condition were achieved in 94% of patients (95% CI, 89–97%; *I*^2^ = 51%). Heterogeneity was obvious with *I*^2^ = 51% and *P* = 0.03 for heterogeneity (Fig. 2 and Table 2).

### Quality assessment

All the included studies were self-controlled trials, which can reflect the outcomes of surgical treatment credibly and the impact on the patients accurately. In the assessment of quality, the mean value of the MINORS score was 13.5 (median, 14; range, 11–15). According to the MINORS score, the quality level of the included studies was overall acceptable, even though none of the studies received a full score on all the items. No study was excluded because of low quality. The MINORS scores are given in Online Resource 2.

### Heterogeneity and sensitivity analysis

Statistical heterogeneity across all the included studies was generally acceptable for most outcomes. Significant heterogeneity with *I*^2^ > 50% was observed in the meta-analysis of total tumor resection (*I*^2^ = 71%), surgical morbidity (*I*^2^ = 56%), and functional improvement at long-term follow-up (*I*^2^ = 51%) (Table 2). Specifically, the heterogeneity for total tumor resection was high with *I*^2^ = 71% if Joseph’s study was included. The sensitivity analysis showed that the heterogeneity for the total resection outcome was reduced to *I*^2^ = 0% after omitting Joseph’s study. Therefore, Joseph’s study may be a cause of significant heterogeneity in the analysis of total tumor resection, and the result should be interpreted with caution. Sensitivity tests were also utilized to inspect the possible causes of between-study heterogeneity for surgical morbidity and long-term neurological function improvement. No factors were recognized as being responsible for the heterogeneity.

### Publication bias

Egger’s test was used to identify possible publication bias. The *P* value > 0.5 for each meta-analysis revealed no evidence of significant publication bias (Table 2).

## Discussion

The incidence of brainstem hemangioblastoma is 2–20% of all intracranial hemangioblastomas, and the most common location is the medulla oblongata. Hemangioblastomas of the brainstem usually occur as solid tumors [39]. Brainstem hemangioblastomas are benign lesions located in complicated anatomical structures, which may cause severe neurological deficits and even death. To date, surgical resection has often been the first option for brain hemangioblastomas that appear to be symptomatic or radiologically progressive. In the past, surgical resection of brainstem hemangioblastomas resulted in poor outcomes due to the complex structure of the brainstems and the rich blood supply of hemangioblastomas. Consequently, a number of patients were referred for radiosurgery. With the advent of microscopy and advances in microsurgical techniques, however, total tumor resection and mortality have become acceptable. Many studies have reported outcomes with respect to the safety and efficacy of brainstem hemangioblastoma surgery. By analyzing all brainstem hemangioblastoma cases from 13 studies, our meta-analysis provided data on the outcomes of surgical treatment and information for neurosurgeons to treat brainstem hemangioblastomas. This study revealed that surgical resection of brainstem hemangioblastomas was reliable, with favorable functional outcomes in 94% of patients. It was technically feasible to remove brainstem hemangioblastomas surgically according to the high rate of total tumor resection (99%) and acceptable rates of postoperative mortality (4%) and neurological morbidity (15%).

As hemangioblastomas are benign and highly vascularized neoplasms with possibilities of recurrence, the primary goal of surgery is radical resection of tumors without significant damage to the surrounding parenchyma. The mortality and complication rates increase markedly with secondary operation on the residual tumors [10, 17]. Neurosurgeons, therefore, always endeavor to remove the tumors completely to avoid tumor recurrence and reoperations, which contributes to the high rate of total resection. To accomplish total resection, it is recommended that surgical management adheres to the principle of arteriovenous malformation surgery, which includes identification and division of the feeding arteries, followed by dissection of the lesion with the preservation of the main draining veins and the occlusion of the main draining veins at the last moment [13, 15, 17, 37, 38, 40]. It is important to perform *en bloc* resection of both solid and cystic lesions, as piecemeal resection may lead to unmanageable intraoperative bleeding. Fully exposing the operative field is critical for intraoperatively locating the lesions and successfully completing the operation. For the dorsal lesions, the suboccipital approach was usually chosen for the craniotomy. The far-lateral approach was performed for lesions located in the ventral part of the brainstem. In particular, C1 and C2 laminectomy were used to enlarge the caudal exposure for lower brainstem lesions if necessary. The pooled proportion calculated in this meta-analysis revealed that total gross resection was achieved in 99% of all cases, indicating that total resection is technically practicable and feasible.

The pooled mortality of all cases was 4%. The result was relatively low but not highly favorable. Although the optimal timing for surgery is debatable, the consensus on the operative intervention among most neurosurgeons is based on the progression of neurologic symptoms or evidence of tumor growth [10, 17, 22, 35, 38]. Therefore, patients treated by surgery were more likely to have giant tumors or unfavorable preoperative functional status that was associated with the high rate of complications and mortality. Furthermore, solid hemangioblastomas usually occur in the brainstem, which raises the risk of parenchyma injuries and consequently increases the mortality [40].

However, compared with the high mortality in the pre-microsurgical era, the mortality in our meta-analysis has significantly improved [4, 38, 40]. Microsurgery at present is generally a safe option for patients with brainstem hemangioblastoma. The improvement of surgical mortality may be attributed to the advent of microscopes as well as advances in surgical techniques, including intraoperative neurophysiological monitoring, preoperative angiography, and embolization [4, 15, 17, 38, 40]. Preoperative angiography contributed to a better understanding of the vascular arrangement and design of the surgical strategy. The role of preoperative embolization and the necessity of intraoperative neurophysiological monitoring, however, remain controversial. Specifically, Wu et al. reported favorable outcomes of 11 patients undergoing preoperative embolization and concluded that preoperative embolization was a safe and effective adjunct treatment [36]. Contrary to his study, many authors insisted that preoperative embolization was likely to cause complications linked to the procedures, such as bleeding, tumor swelling, and vessel occlusion with consecutive infarction.

Seven articles reported the causes of death, among which postoperative pneumonia was a major cause of mortality, followed by intracranial hematoma, brainstem injury, and cardiac infarction or heart failure. Although advances in surgical techniques and instruments have improved surgical outcomes, it remains important to call attention to postoperative complications. The occurrence of postoperative complications varied widely across all the studies. The most common complications included gastrointestinal ulceration or bleeding, pneumonia, lower cranial nerve deficits, hydrocephalus, and CSF leakage. Injury of the dorsal motor nucleus of the vagus nerve may account for the gastrointestinal ulceration or bleeding and respiratory center dysfunction may account for the observed pneumonia. Deficits of the lower cranial nerve usually occurred due to damage to the medulla and manifested as dysphagia and hoarseness. Hydrocephalus and CSF leakage were common complications in the posterior fossa, as reported in the literature [25]. Most of the complications were cured with active and positive treatment. Meticulous microsurgical techniques with adequate postoperative management and care may reduce the incidence of postoperative complications.

Both brainstem lesions and surgical treatment may result in neurological deficits; thus, it is of great significance to assess the functional outcomes of patients with brainstem hemangioblastomas. Comparing preoperative and postoperative scores of McCormick scale or KPS, which has been used in many studies, is considered a practical method to demonstrate the functional outcome of brainstem hemangioblastoma surgery. By analyzing the data on the functional conditions, this meta-analysis revealed that 11% of patients experienced a worse neurological condition immediately after surgery or at discharge, however, most of which were notably transient and usually improved at long-term follow-up. Based on the low morbidity rate and reversible function after surgery, therefore, it seemed a reasonable hypothesis that surgical resection of hemangioblastomas prevents the decline of neurological function. We also considered long-term functional outcomes. The pooled rate of improved functional status at the last follow-up was 85% in our meta-analysis, supporting the evidence that most patients can live and work independently and normally after surgery. It was also observed that favorable outcomes of long-term functional status were generally achieved in a majority of the patients at the last follow-up.

Several factors may have influenced the postoperative outcomes. It is generally agreed that the preoperative functional condition is a good predictor of postoperative neurological function status. Patients with good preoperative function are apt to improve neurologically or remain stable. Furthermore, it was also observed that the patients who had undergone neurosurgical procedures or radiosurgeries were more likely to suffer from the deterioration of neurological function. Some studies suggested that larger tumor or cyst size increased surgical morbidity and mortality, while other studies came to the conclusion that the tumor or cyst size did not affect the results. Despite the controversy regarding the role of tumor size, it is advisable to carefully evaluate the risk of intraoperative bleeding and postoperative complications when treating larger lesions. In particular, Wind et al. considered the presence of syringobulbia as a positive factor for immediate neurological improvement, while Giammattei et al. found brainstem edema a negative prognostic factor. Further investigations are required to confirm the association between these factors and surgical outcome.

Although surgical treatment is a therapeutic approach to cure CNS hemangioblastomas, radiosurgery has a role in the management of hemangioblastomas, especially for patients with nonresectable, residual, and recurrent tumors [14, 31]. Pan et al. analyzed 26 studies on stereotactic radiosurgery for the treatment of difficult-to-resect hemangioblastomas and proposed that stereotactic radiosurgery can serve as a reliable treatment alternative with relatively long-term efficacy in radiographic and symptomatic control. In Pan’s study, the pooled 5-year progression-free survival was 88.43%. The pooled 5-year PFS for patients with intracranial lesions is 88.44%, and with spinal lesions is 90.42%. Factors including solid tumors, smaller tumors, VHL-related lesions, and a greater marginal dose were found to be associated with better tumor control. Adverse events were related to increasing marginal dose, independent of tumor volume [21]. Despite this favorable conclusion, the utility of stereotactic radiosurgery on brainstem hemangioblastomas is limited and requires more study.

## Limitations

Several limitations should be considered in the present meta-analysis, so the interpretation of the results needs to be cautious. Firstly, all the included articles were retrospective and observational studies with inherent limitations, and no randomized clinical studies were included. Furthermore, many of the studies had limited sample sizes, which may have affected the results. Omitting a study when calculating the pooled mortality may have led to an underestimation of the results. Data on functional condition were available for few studies, which limited a definitive conclusion. In addition, only English language studies were included, which may have led to a lack of information from studies published in different languages. Since our study is exploratory, clinicians should use these data for decision-making cautiously.

## Conclusions

In conclusion, while hemangioblastomas are challenging lesions to remove, most brainstem hemangioblastomas can be successfully resected with generally favorable mortality, morbidity, and neurological function. Surgical treatment is a therapeutic management to cure brainstem hemangioblastoma and can create patient lasting benefit and cure. Due to the limited scale of some studies and a lack of randomized trials, however, further investigations are needed to confirm these findings.

## Electronic supplementary material

ESM 1 (PDF 15 kb).

ESM 2 (PDF 65 kb).
