# Supplementary material for: Safety and efficacy of surgical treatment for brainstem hemangioblastoma: a meta-analysis
Source: Neurosurg Rev. 2020 Apr 30;44(2):799–806. doi: 10.1007/s10143-020-01305-3 (PMC8035120; doi:10.1007/s10143-020-01305-3)
Supplement: Supplementary file 1 — (PDF 15 kb). [file 10143_2020_1305_MOESM1_ESM.pdf]

Online Resource 1. The search strategy

Data base	Search strategy
Pubmed	Search ((((((("surgery"[Subheading] OR "surgical procedures, operative"[MeSH Terms] OR "surgery"[All Fields] OR "operation"[All Fields] OR "surgical"[All Fields] OR "operative"[All Fields] OR "operative surgical procedures"[All Fields]))) OR (("microsurgery"[MeSH Terms] OR "microsurgery"[All Fields]))) OR (("craniotomy"[MeSH Terms] OR "craniotomy"[All Fields]))) OR (("neurosurgical procedures"[MeSH Terms] OR ("neurosurgical"[All Fields] AND "procedures"[All Fields]) OR "neurosurgical procedures"[All Fields] OR "neurosurgery"[All Fields] OR "neurosurgery"[MeSH Terms])))) AND (((((((("brain stem"[MeSH Terms] OR "brain stem"[All Fields] OR "brainstem"[All Fields]))) OR (("mesencephalon"[MeSH Terms] OR "mesencephalon"[All Fields] OR "midbrain"[All Fields]))) OR (("pons"[MeSH Terms] OR "pons"[All Fields] OR "pons varolii"[All Fields]))) OR (("medulla oblongata"[MeSH Terms] OR "medulla oblongata"[All Fields])))) AND (((("hemangioblastoma"[MeSH Terms] OR "hemangioblastoma"[All Fields] OR "haemangioblastoma"[All Fields] OR "angioblastoma"[All Fields]))) OR (("von hippel-lindau disease"[MeSH Terms] OR "von hippel-lindau disease"[All Fields] OR "von hippel lindau disease"[All Fields] OR "von hippel-lindau syndrome"[All Fields] OR "von hippel-lindau"[All Fields])))) Filters: English<br>Result: 179<br>Date: Sep 19,2019
Embase	No. Query Results<br>#16. #13 AND #14 AND [english]/lim<br>#15. #13 AND #14<br>#14. #11 AND #12<br>#13. #7 OR #8 OR #9 OR #10<br>#12. #5 OR #6<br>#11. #1 OR #2 OR #3 OR #4<br>#10. 'craniotomy'/exp OR craniotomy<br>#9. 'neurosurgery'/exp OR neurosurgery<br>#8. 'microsurgery'/exp OR microsurgery<br>#7. 'surgery'/exp OR surgery OR operation<br>#6. 'von hippel-lindau disease'/exp OR 'von hippel-lindau disease' OR 'von hippel-lindau syndrome' OR 'von hippel-lindau'<br>#5. 'hemangioblastoma'/exp OR hemangioblastoma OR haemangioblastoma OR angioblastoma<br>#4. 'medulla oblongata'/exp OR 'medulla oblongata' OR 'brain medulla' OR 'medulla oblongatus'<br>#3. 'pons'/exp OR pons<br>#2. 'mesencephalon'/exp OR mesencephalon OR midbrain<br>#1. 'brain stem'/exp OR 'brain stem' OR brainstem<br>Result: 256<br>Date: Sep 19,2019
Web of Science	SET # 4<br>#3 AND #2 AND #1<br>Databases= WOS, BIOSIS, CSCD, DRCI, DIIDW, INSPEC, KJD, MEDLINE, RSCI, SCIELO Timespan=All years

	Search language=Auto<br><br>SET # 3<br>TOPIC: (surg*) OR TOPIC: (operat*) OR TOPIC: (microsurg*) OR TOPIC: (craniotomy) OR TOPIC: (neurosurg*)<br>Databases= WOS, BIOSIS, CSCD, DRCI, DIIDW, INSPEC, KJD, MEDLINE, RSCI, SCIELO Timespan=All years<br>Search language=Auto<br><br>SET # 2<br>TOPIC: (hemangioblastoma) OR TOPIC: (haemangioblastoma) OR TOPIC: (angioblastoma) OR TOPIC: (von hippel-lindau disease) OR TOPIC: (von hippel-lindau syndrome)<br>Databases= WOS, BIOSIS, CSCD, DRCI, DIIDW, INSPEC, KJD, MEDLINE, RSCI, SCIELO Timespan=All years<br>Search language=Auto<br><br>SET # 1<br>TOPIC: (brain stem) OR TOPIC: (brainstem) OR TOPIC: (mesencephalon) OR TOPIC: (midbrain) OR TOPIC: (pons) OR TOPIC: (medulla oblongata)<br>Databases= WOS, BIOSIS, CSCD, DRCI, DIIDW, INSPEC, KJD, MEDLINE, RSCI, SCIELO Timespan=All years<br>Search language=Auto<br>Result: 225<br>Date: Sep 19,2019
