# Supplementary material for: Safety and efficacy of surgical treatment for brainstem hemangioblastoma: a meta-analysis
Source: Neurosurg Rev. 2020 Apr 30;44(2):799–806. doi: 10.1007/s10143-020-01305-3 (PMC8035120; doi:10.1007/s10143-020-01305-3)
Supplement: Supplementary file 2 — (PDF 65 kb). [file 10143_2020_1305_MOESM2_ESM.pdf]

MINORS Scores

| Author | Year | A | B | C | D | E | F | G | H | Score |
|---|---|---|---|---|---|---|---|---|---|---|
| Wang | 2001 | 1 | 2 | 2 | 1 | 1 | 2 | 2 | 0 | 11 |
| Weil | 2003 | 2 | 2 | 2 | 2 | 2 | 2 | 2 | 0 | 14 |
| Zhou | 2005 | 1 | 2 | 2 | 2 | 2 | 2 | 2 | 0 | 13 |
| Pavesi | 2010 | 1 | 2 | 2 | 2 | 2 | 2 | 2 | 0 | 13 |
| Xu | 2010 | 2 | 2 | 2 | 2 | 2 | 2 | 2 | 0 | 14 |
| Wind | 2011 | 2 | 2 | 2 | 2 | 2 | 2 | 2 | 1 | 15 |
| Chen | 2013 | 1 | 2 | 2 | 2 | 2 | 2 | 2 | 0 | 13 |
| Wu | 2013 | 2 | 2 | 2 | 2 | 2 | 2 | 2 | 1 | 15 |
| Yin | 2014 | 1 | 2 | 2 | 2 | 2 | 2 | 2 | 0 | 13 |
| Liu | 2015 | 2 | 2 | 2 | 2 | 2 | 2 | 2 | 0 | 14 |
| Ma | 2015 | 1 | 2 | 2 | 2 | 2 | 2 | 0 | 1 | 12 |
| Giammattei | 2016 | 2 | 2 | 2 | 2 | 2 | 2 | 2 | 0 | 14 |
| Joseph | 2018 | 2 | 2 | 2 | 2 | 2 | 2 | 2 | 0 | 15 |

Items A–H represent: A, a clearly stated aim; B, inclusion of consecutive patients; C, prospective collection of data; D, endpoints appropriate to the aim of the study; E, unbiased assessment of the study endpoint; F, follow-up period appropriate to the aim of the study; and G, loss to follow-up less than 5%; H, prospective calculation of the study size.
